# Eating contexts determine the efficacy of nutrient warning labels to promote healthy food choices

**DOI:** 10.3389/fnut.2022.1026623

**Published:** 2023-01-06

**Authors:** Sara Caballero, Cristóbal Moënne-Loccoz, Mauricio Delgado, Luis Luarte, Yanireth Jimenez, José E. Galgani, Claudio E. Perez-Leighton

**Affiliations:** ^1^Departamento de Fisiología, Facultad de Ciencias Biológicas, Pontificia Universidad Católica de Chile, Santiago, Chile; ^2^Programa de Magister en Nutrición, Facultad de Medicina, Pontificia Universidad Católica de Chile, Santiago, Chile; ^3^Departamento de Ciencias de la Salud, Facultad de Medicina, Pontificia Universidad Católica de Chile, Santiago, Chile; ^4^Programa Interdisciplinario de Neurociencia, Facultad de Medicina, Pontificia Universidad Católica de Chile, Santiago, Chile; ^5^Departamento de Nutrición, Diabetes y Metabolismo, Facultad de Medicina, Pontificia Universidad Católica de Chile, Santiago, Chile

**Keywords:** eating contexts, warning labels, food choice, mouse-tracking, food labels

## Abstract

**Introduction:**

Unhealthy food choices increase the risk of obesity and its co-morbidities. Nutrition labels are a public health policy that aims to drive individuals toward healthier food choices. Chile has been an example of this policy, where mandatory nutrient warning labels (NWL) identify processed foods high in calories and critical nutrients. Eating contexts influence individual food choices, but whether eating contexts also influence how NWL alter the decision process and selection during food choice is unknown.

**Methods:**

In an online mouse-tracking study, participants prompted to health, typical, or unrestricted eating contexts were instructed to choose between pairs of foods in the presence or absence of NWL. Conflict during choices was analyzed using mouse paths and reaction times.

**Results:**

NWL increased conflict during unhealthy food choices and reduced conflict during healthy choices in all contexts. However, the probability that NWL reversed an unhealthy choice was 80% in a healthy, 37% in a typical, and 19% in an unrestricted context. A drift-diffusion model analysis showed the effects of NWL on choice were associated with an increased bias toward healthier foods in the healthy and typical but not in the unrestricted context.

**Discussion:**

These data suggest that the efficacy of NWL to drive healthy food choices increases in a healthy eating context, whereas NWL are less effective in typical or unrestricted eating contexts.

## 1. Introduction

The modern human food environment provides access to a variety of foods, including those of high palatability and caloric content (1–3). The larger reinforcing effects of calorie-dense food favor their choice over less calorie-dense food, thereby increasing calorie intake and the risk of obesity (4–7). Thus, our environment often introduces the conflict between choosing unhealthy but more palatable foods or healthier but less palatable foods (8–10). Understanding how food choice unfolds and how to influence individuals to select healthy foods is necessary to design better or improve current strategies promoting healthy food choices.

**FIGURE 1:**
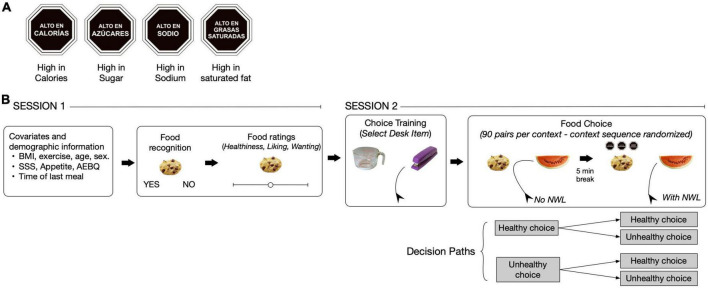
Nutrient warning labels (NWL) and experimental design. **(A)** NWL used in the experiment. At the bottom of each label is the English translation from the original Spanish. **(B)** The experiment had two sessions. Demographic information and subjective ratings were collected in the first session and the food choice tasks were conducted in the second session.

Food labeling is a public health strategy that aims to promote healthier food choices (11). Food labels used worldwide vary in the information displayed (e.g., nutrient information or a single descriptor), the symbology used (e.g., numbers or traffic lights), whether the information requires being interpreted by the user (e.g., indicating the amount of fat in a product or if the product is high in fat) and if the label conveys a positive or negative message (e.g., this product is healthy or unhealthy) (11, 12). Nutrient warning labels (NWL) are a type of label that indicates whether a food item is high in calories or critical nutrients (13). NWL can increase the purchase of healthy food, reduce the purchase of unhealthy food, and reduce the overall energy content purchased (13). Chile is a prominent example of the use of NWL, where since 2016, mandatory NWL on processed foods indicate whether a food is high in calories, saturated fat, sugar, or sodium (14–16; Figure 1A). Survey and focus group studies suggest that NWL changed the perception of and attitude toward food conducive to healthier food choices (17–20). Consistently, and compared to the counterfactual condition where NWL were not implemented, the purchase of foods with NWL was reduced 2 years after their implementation. Still, in the same period, the purchase of foods without NWL increased, thereby reducing total energy purchased only by 16.4 kcal/capita/day (21, 22). Despite the widespread use of NWL in Chile and other countries, how NWL modify the decision process and its outcome during food choice remains unexplored.

Food choice is a multifactorial process (8), and the concept of eating context describes a subject’s environment and internal state during food choice (23, 24). For example, a healthy eating context in a real-world setting would be created by combining a lower price, increased information, and easier access to healthy food (25). In experimental studies, the eating context is often defined by explicit instructions highlighting a particular aspect of a real-world context before a food choice task. As such, an experimental healthy eating context can be created by prompting participants to consider the healthiness of food or its health consequences. This intervention reduces the portion size and probability of choosing unhealthy foods compared to participants prompted to select foods based on taste or desire (26–32). The effects of a health context on food choice include increasing the value of health-related food attributes over others like taste (27, 33). These effects are also consistent over time (34) and correlate directly with dietary self-control-related brain activity (29). Thus, eating contexts influence food choice and can be implemented in laboratory settings to study the determinants of food choice.

There is scarce evidence on whether eating contexts alter the impact of food labels on food choice. Eye-tracking studies suggest that the eating context influences engagement with food labels and their effect on choice, but whether this translates to experimental or real-world choices is unclear (35). For example, food labels appear to be more effective among those with high subjective health and nutrition knowledge (36). However, others have reported that these characteristics increase time spent looking at labels but do not alter choice (37). Also, one study suggests that external contexts (i.e., shopping vs. home) do not influence whether food labels are read (38). Regarding NWL, only one cross-sectional survey showed that context during purchase, described as having a child requesting high-sugar or high-fat foods, reduced the effect of NWL on decreasing the purchase of foods high in sugar (39). Thus, whether the eating context influences the effect of NWL food choice remains largely unexplored.

Our goal was to identify whether the presence of NWL could affect the decision process and food choices across different eating contexts. To this end, we conducted an online study where participants were prompted to a healthy, typical, and unrestricted eating context to choose between pairs of food images of different healthiness in the absence and presence of NWL.

## 2. Materials and methods

### 2.1. Rationale of the approach

Figure 1B summarizes the experimental approach. Participants were asked to choose a food item from pairs of food images of different healthiness and palatability in three previously used contexts (31): Health, typical, and unrestricted. In the healthy context, participants were instructed to select the food they should eat to be healthy, which aimed to reflect their ideal of a healthy diet. In the typical context, participants were instructed to select as they would do in their daily life, which aimed to reflect daily food choices (31). In the unrestricted context, participants were instructed to choose as if nothing was stopping them, which aimed to reflect situations where health concerns are minimized (i.e., dining out for pleasure). The healthy context was included to facilitate interpreting choice data, as this context drives healthier food choices compared to other contexts (26–31). Using different sets of food images in each context, we measured the probability of a healthy choice and conflict during food choice first without showing NWL for all pairs of food images and then showing NWL for the same pairs. This design created paths for each decision (Figure 1B) to test whether showing NWL would reverse unhealthy food choices made in the absence of NWL across the eating contexts. For all food choices, we estimated conflict by measuring the area under the curve (AUC, the difference in the mouse path from the direct line between the starting point and the chosen image) and reaction time (RT). A larger AUC and longer RT are interpreted as a larger conflict during choice (40–43). Finally, to gain insight into how NWL and context affect the decision process, we fitted a drift-diffusion model (DMM) to the choice data to examine whether changes in AUC could be related to changes in drift rate (how quickly a subject accumulates evidence toward a healthy choice) and decision bias (baseline probability of choosing a given option) toward healthier foods.

### 2.2. Participants

The Human Ethics Committee at Pontificia Universidad Católica de Chile approved this study (Protocol 201223001). Participants were recruited through social media (Instagram and Facebook) and email between June and November 2021 as a convenience sample (the only consideration was to recruit an equal number of males and females by biological sex) and offered a local retailer’s gift-card for completing the study (Cencosud, 20,000 Chilean pesos, ∼25 USD). Inclusion criteria were BMI between 20 and 35 kg/m^2^, 18–45 years of age, absence of any disease, and stable body weight (less than 2 kg change over the last 3 months). Exclusion criteria were undergoing treatment for body weight loss, consuming any medication or nutritional supplement, tobacco or alcohol use, any eating disorder or restrictive dietary style, professional sports activity, pregnancy, or breastfeeding. Supplementary Figure 1 describes the workflow for participant selection. Briefly, participants that contacted investigators were sent a screening questionnaire on a rolling basis. Of the 716 individuals that received the screening questionnaire, 686 returned it. Of those, 496 were excluded, 181 were invited to the study, and 149 completed it. The main rejection criteria were the use of nutritional supplements (*n* = 230), weight change during the last 3 months (*n* = 138), and dietary restrictions (*n* = 128), with 66% of the individuals excluded based on any of these criteria. Among the 149 participants that completed the study, we eliminated 14 participants due to inconsistencies in demographic responses between the screening questionnaire and data collected during the study, one for completing the study twice, one for reporting not reading instructions, one for taking more than 3 hours in completing a single session, and two for having less than 2% of the mouse-tracking data recorded. Thus, data from 128 participants were considered for analysis.

### 2.3. Experimental design

The experiment was implemented in Spanish on the Gorilla Online Platform (44). Figure 1B summarizes the experimental design (Supplementary Table 1 details all the steps in the study). After screening, participants were instructed to complete the two sessions of the study at the same time of day within seven days. In the first session, participants completed a questionnaire for demographic information, exercise, the same questions from the screening questionnaire (body weight, height, age, variation of body weight in the last 2 months, dietary restrictions, alcohol and tobacco use, see Supplementary Table 2 for a Spanish and English version of the questionnaire), and the time of their last meal. Next, participants completed the Sleep Stanford Scale [SSS (45)], answered four questions about hunger and appetite using visual analog scales (VAS) from 0 to 100 (Questions 1−4 from Supplementary Table 3; 46), and the Adult Eating Behavior Questionnaire (AEBQ) (47). The SSS is a one-question form that measures the perceived level of sleepiness and was included to control for potential effects of sleepiness on the desire for high-calorie foods (48). The questions about hunger and appetite were included to control for appetite effects of choice as increased appetite measured by VAS is a predictor of calorie intake and can influence a subject toward choosing high-calorie foods (46, 49). The AEBQ measures different aspects of food approach and avoidance and has been validated in a sample of Chilean adults (47, 50) and was included to control for potential effects of eating behavior on food choice (51).

To complete the first session, the participants answered whether they recognized 94 food images and then were asked to rate those images for healthiness, liking, and wanting using a VAS scale from 0 to 100 (52) (Question 5–7 from Supplementary Table 3).

In the second session, participants recorded the time of their last meal and answered the SSS and the same questions about hunger and appetite as in the first session. Next, participants completed a training task instructing them to choose the desk item between images of a desk and a kitchen item (50 pairs of images). Next, participants were randomized to one of six possible sequences of contexts (typical, healthy, unrestricted) for the food choice task. In each context, participants had to choose one food image from 90 pairs without displaying the NWL. After a 5 min break, the same procedure was repeated, but now the NWL were show for each food of the same 90 pairs. Participants also had a 5-min break between contexts. Finally, participants completed a survey where they selected the option that best described how they used NWL in the task (counted the number of NWL, read the information displayed by NWL, or did not use NWL).

### 2.4. Food images

Food images (FoodPics database) (53) were classified into the following categories: bread (*n* = 3), breakfast food (*n* = 4), cake (*n* = 9), cheese (*n* = 3), cookie (*n* = 5), dessert treats (*n* = 22), fruit (*n* = 25), pasta (*n* = 4), pastries (*n* = 7), pie cakes (*n* = 1), pizza (*n* = 3), prepared meals (*n* = 28), salads (*n* = 3), seafood (*n* = 1), seeds nuts (*n* = 6), snacks (*n* = 14), soups stews (*n* = 5), and vegetables (*n* = 24). Online nutritional information for each food image was used to assign NWL following the Chilean Food Labeling Law (14) (See Figure 1A for the NWL used in the study). A pilot test (*N* = 8 adult subjects) indicated that 18 images were not recognized and thus were removed from the dataset. We also selected ten images of typical Chilean preparations assigned to the categories of prepared meals and dessert treats that were validated for recognition in a separate pilot test (*N* = 6). All food images (*N* = 94, see Supplementary Information for detailed information including food pictures) were assigned with 0 to 3 NWL as foods with 4 NWL (high in calories, sugar, saturated fat, and sodium) were infrequent. Images were assigned to each context following a stratified sampling strategy from different food categories. The health context included 32 images (0 NWL: 8, 1 NWL: 6, 2 NWL: 8, 3 NWL: 10), the typical context 29 (0 NWL: 7, 1 NWL: 6, 2 NWL: 7, 3 NWL: 9), and the unrestricted context 33 images (0 NWL: 8, 1 NWL: 6, 2 NWL: 8, 3 NWL: 11). The calories, saturated fat, sugar, and sodium were not different among images used in the different contexts (Supplementary Figure 2 and Supplementary Table 4).

### 2.5. Eating contexts

The eating contexts were defined by the instructions given to participants (31). In the healthy eating context, participants were instructed to “select as fast as possible the food that you would eat to stay healthy,” in the typical eating context to “select as fast as possible the food that you would usually select in your daily life,” and in the unrestricted eating context to “select as fast as possible the food that you would eat if nothing stopped you.”

### 2.6. Food choice task

Each trial started with a white screen that displayed a button labeled “START” at the bottom center of the screen. Once participants pressed the start button, they had up to four seconds to select between food items that appeared in the upper left and right corners with or without NWL displayed. After clicking on the selected food image, a white screen with a fixation cross displayed for a randomly selected period between 100 and 500 ms before the next pair of images was presented. For each participant, the image pairs used in each context were generated pseudo-randomly as follows: (1) Each participant saw 15 food image pairs for each of the six possible combinations of numbers of NWL per food image (0 vs. 1, 0 vs. 2, 0 vs. 3, 1 vs. 2, 2 vs. 3 NWL); (2) Any food image had to skip two pairs before being shown again; (3) Food images with the same number of NWL were not shown on the same side for more than 3 consecutive pairs. Food images were not repeated between contexts to prevent participants from recalling NWL associated with each image.

### 2.7. Data analysis

#### 2.7.1. Data preparation

For each choice, we computed the AUC (difference between the actual and a straight trajectory between the starting point and selected image) and the number of y-axis crossings using the mousetrap R package (54). Food choice trials were selected for analysis following standard recommendations for data quality (41, 55). Trials not considered had a mouse-tracking resolution lower than 10 Hz (3.63% total trials); > 3 standard deviations (SD) above the participant’s mean for initiation time (0.83% total trials), AUC (0.91% total trials), or RT (2.00% total trials); the y-axis was crossed three or more times (2.45% total trials), and none or only one food image was recognized (17.89%).

#### 2.7.2. Drift diffusion model (DDM)

Drift diffusion models for each choice trial were fitted using the RWiener package. To test for model fit, we followed a procedure based on Monte-Carlo simulations (56). For each combination of context and presence of NWL we draw 1,000 parameter sets from a multidimensional normal distribution (mvtnorm R package) using the means and covariance matrix calculated from the individual fitted parameters DDM (decision boundary, non-decision time, decision bias, or starting point, and drift rate). We simulated a sample of 128 RT for each parameter set that was used to re-fit the DDM as done in the original data set, creating a dataset of recovered parameters. Any individual level parameters lying outside the 5% quantile of the distribution of recovered parameters were excluded from the analysis. Also, we computed the correlation coefficient between recovered parameters and the empirical fit to assess if parameters were successfully recovered.

### 2.8. Statistical analysis

All analyses were adjusted for covariates. The covariates were trial order during the task (numeric), age (numeric), sex (male/female), body mass index (BMI, numerical), SSS score (numerical), AEBQ score (numerical) (47), physical exercise (yes/no), appetite score (the mean of the four questions about hunger and appetite, see Supplementary Table 1), hour of test start (numerical) and time since last meal (hour, numerical). All continuous covariates were centered and scaled. For all linear mixed models, participant was included as random effect, and each term’s statistical significance was calculated using Wald Type III ChiSquare test. Adjusted estimates and posthoc tests for dependent variables were done on estimated marginal means (emmeans R package). The mixed effects logistic regression (glme function) and linear mixed models (lme function) functions were from the lme4 R package. All results are presented as mean and standard error. Post-hoc comparisons were corrected by Tukey’s Honest Significant Difference.

#### 2.8.1. Effect of NWL on subjective health, like, and want ratings

The response variables were the VAS score for health, like, and want ratings for each image in a linear mixed model with the number of NWL for each image as fixed effect.

#### 2.8.2. Demographic parameters

Differences between males and females were tested using *t*-tests for numeric variables and Chi-square test for frequency variables.

#### 2.8.3. Differences in food attributes across eating contexts

The response variables were calories, saturated fat, sugar, and sodium per 100 g in a two-way ANOVA with the interaction between NWL and context as independent variables.

#### 2.8.4. Effect of eating contexts on the probability of selecting a healthy food without showing NWL

The response variable was the probability of a healthy choice (whether the food with the lower number of NWL was selected) in a generalized mixed logistic regression with the two-way interaction between contexts (healthy, typical, unrestricted) and difference in health and like ratings as fixed variables. The difference in the health and like ratings were calculated by subtracting the rating of the selected image from the rating of the non-selected image for each trial and participant.

#### 2.8.5. Effect of eating context on AUC and RT during food choice without showing NWL

The dependent variables were AUC and RT in a linear mixed model with the interaction between food choice (healthy vs. unhealthy) and context as fixed effects.

#### 2.8.6. Effect of showing NWL on the probability of healthy food choice

The response variable was the probability of a healthy choice (whether the food with the lower number of NWL was selected) in a mixed logistic effects regression with the two-way interaction between context (healthy, typical, unrestricted) and the food choice without NWL (healthy vs. unhealthy) as fixed effects.

#### 2.8.7. Effect of eating context and presence of NWL on change in AUC and RT during food choice

Changes in AUC (ΔAUC) and RT (ΔRT) were calculated as the difference in each variable between the first choice (NWL not shown) and the second choice (NWL shown). Thus, a negative value indicated a reduction in either AUC or RT caused by NWL. The dependent variables were ΔAUC or ΔRT corrected for the baseline value (AUC or RT during the choice without NWL) in separate linear mixed models with the two-way interaction between eating context and decision path (the four combinations of choices, Figure 1B) as fixed effects.

#### 2.8.8. Effect of eating context and showing of NWL on DDM parameters

The response variables were bias, drift, and non-decision time in a linear mixed model that included the interaction between context and presence of NWL.

## 3. Results

### 3.1. Participants

Participants were balanced by sex with an average BMI of 23.3 ± 0.2 kg/m^2^ (all participants that completed the study had a BMI < 30) and 24.8 ± 0.5 years old (Table 1). Men and women did not differ in age (*P* = 0.79), BMI (*P* = 0.33), frequency of physical exercise (*P* = 0.89), percent of overweight participants (*P* = 0.28), and education level (*P* = 0.59).

**TABLE 1:** Participant demographic characteristics.

	All	Female	Male
Number of participants	128 (100%)	66 (51.6%)	62 (48.4%)
Age (years)	24.78 ± 0.51 (18–45)	24.65 ± 0.7 (18–45)	24.92 ± 0.74 (18–43)
BMI (body mass index)	23.25 ± 0.2 (19.49–29.39)	23.06 ± 0.27 (20.05–29.38)	23.46 ± 0.31 (19.49–29.39)
Normal weight (%) (BMI between 19.5 and 24.9)	80.5% (103)	84.8% (56)	75.8% (47)
Overweight (%) (BMI between 25 and 30)	19.5% (25)	15.2% (10)	24.2% (15)
Adult eating behavior questionnaire (AEBQ) score	1.33 ± 0.05 (0.58–3.61)	1.27 ± 0.06 (0.64–3.61)	1.4 ± 0.07 (0.58–2.88)
Physical exercise (yes/no)	68.0% (87)	66.7% (44)	69.4% (43)
**Highest education level completed**			
College	38.3% (49)	34.8% (23)	41.9% (26)
High school	53.9% (69)	57.6% (38)	50.0% (31)
Post-graduate	7.0% (9)	6.1% (4)	8.1% (5)
Primary	0.8% (1)	1.5% (1)	0.0% (0)

### 3.2. Subjective health, like, and want ratings of food images made without showing NWL correlate with the actual number of NWL in images

**FIGURE 2:**
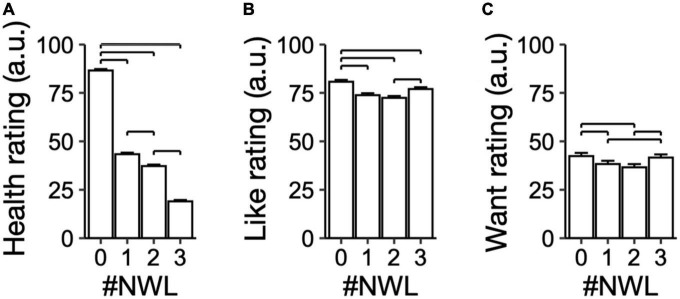
Subjective health, like, and want ratings made without showing nutrient warning labels (NWL). Visual analog scales (VAS) ratings for subjective **(A)** Health, **(B)** Like, and **(C)** Want for all foods used in the study made in the absence of NWL. Foods were grouped based on the actual number of NWL of foods. Brackets, *P* < 0.05 for pairwise comparisons.

Health ratings decreased as the actual number of NWL per food increased (*X_3* = 12052.16, *P* < 0.01; Figure 2A). Like and want ratings had a U-shape as decreased from foods without NWL to foods with 2 NWL and increased to foods with 3 NWL (Like: *X_3* = 184.55, *P* < 0.01; Want: *X_3* = 85.81, *P* < 0.01; Figures 2B, C). Among covariates, increasing BMI reduced health (β = −0.73 ± 0.27, *P* = 0.01) and like ratings (β = −1.05 ± 0.37, *P* < 0.01) but had no effect on want ratings (*P* = 0.84). Supplementary Figure 3 shows the estimates for health, like, and want ratings separated by demographic variables. Overall, participants recognized objectively healthier foods (based on the number of NWL per food image) and showed higher like and want ratings for foods with 0 and 3 NWL.

### 3.3. Eating contexts determine the probability of healthy choices and conflict during food choices made without showing NWL

**FIGURE 3:**
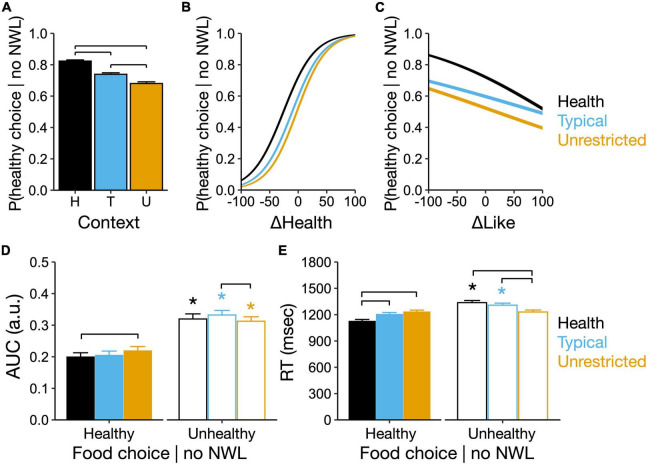
Eating contexts determine the probability of healthy choices and the magnitude of conflict during food choices made in the absence of nutrient warning labels (NWL). **(A)** Probability of healthy food choice made without showing NWL by context. Brackets, *P* < 0.05 for pairwise comparisons. **(B)** Larger differences in subjective health rating (fixing like differences at 0) between food images increase the probability of a healthy choice made without showing NWL in all contexts. **(C)** Larger differences in subjective like rating (fixing health differences at 0) between food images reduce the probability of healthy choices made without showing NWL in all contexts. For panels **(B,C)**, differences in health and like ratings were calculated relative to the chosen food image. **(D)** AUC and **(E)** RT during healthy and unhealthy food choices made without showing NWL. Brackets, *P* < 0.05 pairwise differences between contexts within healthy and unhealthy choices. Asterisks, *P* < 0.05 between healthy and unhealthy choices within contexts.

When participants had to choose between pairs of food images in the absence of NWL, the probability of a healthy choice (choosing the food item with the actual lowest number of NWL between the two options) was 82.4, 73.9, and 68% in the health, typical, and unrestricted contexts respectively (Figure 3A; main effect of context: *X_2* = 286.76, *P* < 0.01; *P* < 0.05 between all contexts). Among covariates, an increase in one unit of BMI increased the probability of a healthy choice by 8.3 ± 3.9% (*P* = 0.01), while appetite had no significant effect (Supplementary Tables 5, 6 show the complete output of the logistic regression). In all contexts, the probability of a healthy choice increased as the difference in health ratings between images increased (βΔHealth ratings = 3.86 ± 0.05%, *P* < 0.01; Figure 3B) and decreased as the difference in like ratings between images increased (βΔLike ratings = −0.06 ± 0.005%, *P* < 0.01; Figure 3C). Overall, in the absence of NWL, the probability of a healthy food choice was higher in the healthy context and lower in the unrestricted context.

There were significant interactions between food choice type (healthy vs. unhealthy) and context for AUC and RT (AUC: *X_2* = 10.52, *P* < 0.01; RT: *X_2* = 242.47, *P* < 0.01; Figures 3D, E). In a healthy choice, the AUC was largest in the unrestricted context and larger compared to the health context (9.7 ± 3.8%, *P* = 0.01, Figure 3D). For an unhealthy choice, the AUC in all contexts was larger compared to the healthy choice (P < 0.05, Figure 3D), and AUC was now largest in the typical context and different from the unrestricted context (larger by 5.9 ± 2.5%, *P* = 0.01, Figure 3D). For RT, healthy choices were slower in the unrestricted context (*P* < 0.05 compared to all contexts, Figure 3E); but unhealthy choices were slower compared to healthy choices only in the health and typical contexts (*P* < 0.05, Figure 3E). Overall, healthy choices in the unrestricted context showed higher AUC and were slower than in other contexts. Compared to healthy choices, unhealthy choices had higher AUC in all contexts and were slower only in the health and typical contexts.

### 3.4. Eating context and choices made in the absence of NWL determine the effect of showing NWL on conflict and decision during food choice

**FIGURE 4:**
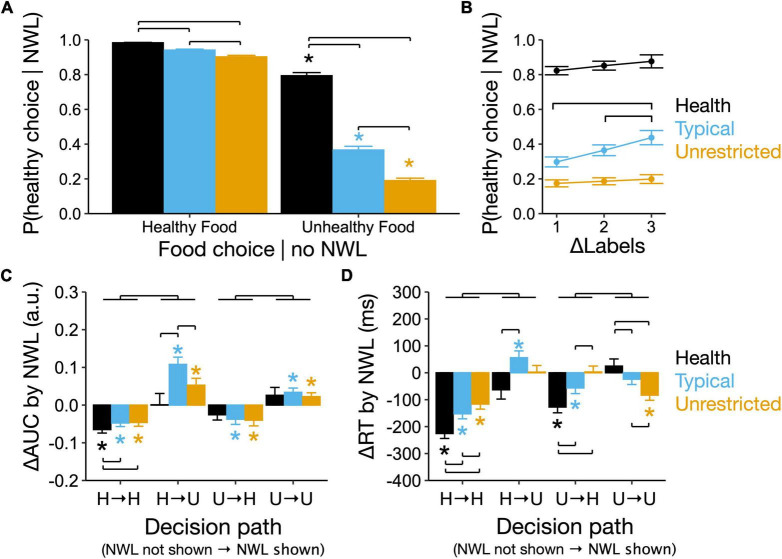
Eating contexts determine the effect of showing nutrient warning labels (NWL) on conflict during decision and outcome of food choice. **(A)** Probability of a healthy food choice when showing NWL based on the prior choice made in the absence of NWL. Brackets, *P* < 0.05 pairwise differences between contexts within healthy and unhealthy choices. Stars, *P* < 0.05 between healthy and unhealthy choices within contexts. **(B)** Effect of increased absolute difference in the number of NWL between foods on the probability of healthy food choices in different contexts. Brackets, *P* < 0.05 pairwise differences between the number of food labels within context. **(C)** Change in area under the curve (AUC) (ΔAUC) and **(D)** change in reaction time (RT) (ΔAUC) between choices made with and without showing NWL. Brackets, *P* < 0.05 pairwise differences between contexts for each decision path (H, Healthy food choice; U, Unhealthy food choice). Asterisks, *P* < 0.05 for ΔAUC or ΔRT being different from zero. Upper brackets, *P* < 0.05 for differences between decision paths averaged over contexts.

The probability of a healthy choice when NWL were shown was higher than 90% in all contexts when participants first made a healthy choice in the absence of NWL (Figure 4A) and was lower in all contexts when their first choice was unhealthy (*P* < 0.05 for pairwise comparisons within contexts across decision paths, Figure 4B). Thus, the probability that showing NWL would reverse an unhealthy choice was 79.6 ± 1.8, 36.6 ± 2.1, and 18.6 ± 1.4% in the health, typical, and unrestricted contexts, respectively (*P* < 0.05 between contexts), and regardless of how participants declared to use NWL during food choices (Among participants, 51% declared to count NWL, 37.5% to read them, and 10.9% to not use them during choice, Supplementary Figure 4). Increasing ΔNWL (the difference in the number of NWL between foods in each pair) increased the probability of a healthy choice only in the typical context (up to 13.5 ± 3.3% for ΔNWL = 3, *P* < 0.05, Figure 4C), regardless of how participants declared to use NWL during food choices (Supplementary Figure 5). Overall, showing NWL had a probability to reverse an unhealthy choice of 80% in the healthy context and lower than 50% in the typical and unrestricted contexts.

The effect of showing NWL on the change in AUC (ΔAUC) and RT (ΔRT) compared to the choice made without showing NWL depended on the decision path (interaction between decision path and context: ΔAUC, *X_6* = 14.36, *P* < 0.01; ΔRT, *X_6* = 140.58, *P* < 0.01). There were no significant effects of BMI, appetite score, or time from the last meal on ΔAUC or ΔRT (Supplementary Table 7). The confirmation of a healthy choice made when NWL were shown was associated with reduced AUC and faster decisions (negative ΔAUC and ΔRT) in all context, and these effects were the largest in the healthy context. Making an unhealthy choice in the presence of NWL, either by reversing a healthy choice or confirming an unhealthy choice made without NWL, was associated with increased AUC (positive ΔAUC) in the typical and unrestricted contexts. Still, these decisions were slower (positive ΔRT) in the typical context and faster (negative ΔRT) in the unrestricted context. Finally, in the presence of NWL, the reversal of an unhealthy food choice made without NWL was associated with reduced AUC (negative ΔAUC) in the typical and unrestricted contexts but faster decisions (negative ΔRT) only in the health and typical contexts (Figures 4C, D).

Overall, compared to decisions made in the absence of NWL, showing NWL reduced the AUC during healthy choices and increased the AUC during unhealthy choices across eating contexts. The reversal of unhealthy food choices by NWL in the healthy context (80% probability) is associated only with faster choices, in the typical context (36% probability) is associated with reduced AUC and faster choices, and in the unrestricted context (14% probability) is associated only with reduced AUC.

### 3.5. Eating contexts determine the effect of showing NWL on decision bias, drift rate and non-decision time during food choice

**FIGURE 5:**
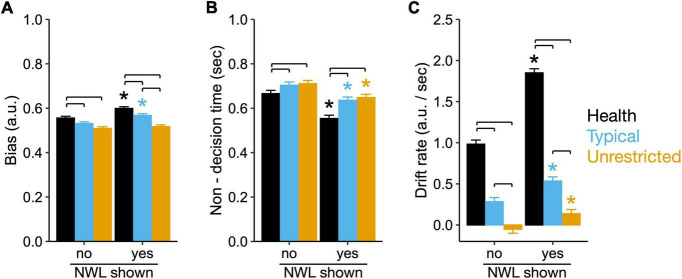
Eating contexts determine the effect of showing nutrient warning labels (NWL) on the bias, non-decision time, and drift rate during food choice. **(A)** Bias, **(B)** Non-decision time, and **(C)** Drift rate derived from a drift-diffusion model (DDM) considering healthy food choices as the upper boundary for food choices made without showing and then showing NWL. Brackets, *P* < 0.05 pairwise differences between contexts for choices made with and without showing NWL. Asterisks, *P* < 0.05 in bias, non-decision time, and drift between choices made with and without showing NWL within context.

There was an interaction between context and presence of NWL for decision bias (*X*_*2*_ = 6.97, *P* = 0.01), drift rate (*X_2* = 76.19, *P* < 0.01) and non-decision time for the healthy choice (*X_2* = 13.08, *P* < 0.01). Regardless of the presence of NWL, the healthy context showed the largest decision bias, drift rate, and shorter non-decision time toward a healthy choice (Figure 5). The unrestricted context showed the opposite pattern (i.e., the smallest bias, drift rate, and longer non-decision time toward a healthy choice, Figure 5). Compared to the choice made without NWL, the presence of NWL increased decision bias for a healthy choice in the health (7.6 ± 1.8%, *P* < 0.01) and typical (6.9 ± 1.9%, *P* < 0.01), but not the unrestricted context (*P* = 0.45; Figure 5A). Also, the presence of NWL reduced non-decision time in all contexts with the largest reduction in the health context (−16.8 ± 1.6%, *P* < 0.01) compared to the typical (−9.6 ± 1.5%, *P* < 0.01) and unrestricted context (−8.8 ± 1.5%, *P* < 0.01; Figure 5B). Finally, the presence of NWL almost doubled the drift rate in the healthy (88.3 ± 6.2%, *P* < 0.01) and typical contexts (89.1 ± 21.8%, *P* < 0.01) and increased it by threefold in the unrestricted context (367.5 ± 117.9%, *P* < 0.01), but the drift rate remained highest in the health context and lowest in the unrestricted context (Figure 5C). Overall, compared to a typical and unrestricted context, a healthy context reduces non-decision time, increases decision bias and drift rate toward healthier foods. NWL reduce non-decision time and increase drift rate toward healthier foods in all contexts but increase bias toward healthier foods only in the health and typical contexts.

## 4. Discussion

This study aimed to understand how eating contexts and the presence of NWL influence food choice. We present three key findings. First, eating contexts determine the probability that NWL can reverse an unhealthy choice, this being over 80% in a healthy context, dropping to 37% in a typical and 19% in an unrestricted context. Second, NWL reduce conflict (as shown by reduced AUC and faster RT) during healthy choices and increase conflict during unhealthy choices. Third, NWL increase healthy food choices in healthy and typical contexts, likely by increasing bias and speed of decision toward healthier foods.

Our data show that in the absence of NWL, the probability of a healthy choice decreases from the healthy to typical and is lowest in the unrestricted context, a finding consistent with others (26–31). The unrestricted context had the largest AUC and longer RT during healthy choices, suggesting the higher conflict is associated with a longer time necessary to consider less salient food attributes (i.e., healthiness) to make a healthy choice in this context (57, 58). As anticipated, in the healthy context, the healthier choices were easier (lower AUC and shorter RT) than the unhealthy choices. We anticipated that an unrestricted context would decrease conflict during unhealthy choices, as we expected participants to favor selecting the less healthy but more palatable foods. Still, the unhealthy choices in the unrestricted context had a larger AUC than healthy choices. Our participants assigned similar like and want ratings to the objectively healthiest foods (those with zero NWL) and less healthy foods (those with 3 NWL). Thus, the increased AUC could be due to participants having similar hedonic ratings for foods of different healthiness. The similar RT for healthy and unhealthy choices in the unrestricted context suggests that participants only consider the most salient attributes of foods (reflected in like and desire ratings) in the decision process. Overall, these data show that in the absence of NWL, a healthy context facilitates healthy choices compared to a typical and unrestricted context.

Our data shows that eating contexts alter the influence of NWL on the decision process and choice. While showing NWL led to a probability of over 90% of confirming the healthy choice made in the absence of NWL across all contexts, the probability that showing NWL would reverse an unhealthy choice was 80% in the health, 37% in a typical, and 19% in the unrestricted context; all regardless of how participants used NWL in their decision. The presence of NWL facilitate healthier choices, as NWL reduced AUC and RT in all contexts during healthy choices; and NWL make unhealthy choices more difficult, as NWL increased AUC in all contexts during unhealthy choices compared to choices made in the absence of NWL. However, the magnitude of the effects was dependent on context. For example, we observed the largest reduction in AUC and RT in the healthy context, which matches retail data indicating that subjects that intend to eat healthy will use nutritional information (59). However, during the reversal of unhealthy choices, NWL reduce AUC in all contexts but RT only in the healthy context. Our DDM analysis provides an insight into this effect. The healthy context has the largest drift rate (how quickly a subject accumulates evidence toward a healthy choice) and bias for healthy food choices (larger bias indicates less information is needed to choose the healthy option). Further, while showing NWL increase drift rate in all contexts, bias for healthier food options only increased in the healthy and typical contexts. Together, these data first suggest that context determines a baseline decision bias and drift rate for healthy choices (higher in the healthy context and lowest in the unrestricted context). Second, that showing NWL increase the probability of healthy choices by reducing conflict during healthy choices and increasing conflict unhealthy choices. This effect of NWL is associated with increased drift rate and bias towards healthier food choices, the latter being active before the decision process and absent in the unrestricted context.

The proposed model fits with evidence that a higher drift rate and an earlier entry into the decision process of health over taste ratings increase the probability of healthy food choices (58). While we did not model the explicit contribution of differences in healthiness and wanting in different contexts in our DDM, we predict that compared to when NWL are absent, displaying NWL would increase drift rate, reduce time of entry into the decision process of healthiness ratings, and bias the decision toward the healthier food. Further, we propose this bias might be mediated by increasing salience of negative health consequences of intake (13). We did not include the monetary cost of food, which is relevant as NWL are expected to drive healthier choices despite the higher price of healthier foods often limits their purchase (60–62). Thus, future studies should also examine whether explicit information about monetary cost influences the effect of context and NWL on food choices.

Limitations of our study include potential selection bias and food choice conditions that differ from real-world conditions. Our participants were selected as a convenience sample, which could influence our results. For example, the incidence of obesity in Chile is over 34% (63). Still, there are no participants with obesity in our study. Thus, the healthy context of our participants (what they would eat to be healthy) could be closer to their typical behavior compared to participants with obesity. This might explain that while the probability that showing NWL reverses an unhealthy choice is above 50% only in the healthy context, showing NWL reduces conflict during the reversal of unhealthy choices in healthy and typical contexts. The artificial nature of food choice in our study is a limitation inherent to experimental studies of human behavior. For example, our participants had up to 4 s to choose a food image between two options. While these constraints can increase attrition during the test and simplify data analysis, food choices are not binary or time constrained in a real-world setting. Overall, future studies using larger samples should address the potential impact of obesity (and other factors such as sedentarism or age) in food choice and NWL under different contexts in more complex experimental designs that better model real-world conditions.

Despite its limitations, we think these data have strong implications for public health interventions. Analysis of purchase data indicates that NWL increased the selection of foods without NWL. Still, the resulting reduction in calories purchased is low (21, 22) and within range of what can be compensated by reducing energy expenditure to maintain a stable body weight (64). Our data suggest that prompting a healthy context before or during food purchase could increase the efficacy of NWL to drive healthier food choices and have a larger impact on calorie intake compared to typical behavior and to situations of unrestrained eating where health considerations are minimized (i.e., dining out, buying food for pleasure). Most interventions at food retailers or restaurants have focused on improving the healthiness of the food available and increasing the visibility of healthier options (65, 66). Still, interventions that prompted a healthy context in cafeteria settings led to increased dietary quality (67, 68), which might increase further if NWL are included in the intervention. Thus, our data suggest that interventions that prompt healthy eating contexts during purchase could improve the efficacy of NWL to drive healthier food choices.

In conclusion, NWL can facilitate healthier food choices and make unhealthy choices more difficult in a healthy, typical, and unrestricted context. Still, their ability to reverse an unhealthy food choice is below 50% in a typical and unrestricted context. Our DDM analysis suggests this effect is likely determined by information considered before the start of the decision process, which might be associated with negative health consequences triggered by NWL. These data suggest that private or public prompts for a healthy eating context could increase the ability of NWL to drive healthier food choices.

## Data availability statement

The raw data supporting the conclusions of this article will be made available by the authors, without undue reservation.

## Ethics statement

The studies involving human participants were reviewed and approved by Human Ethics Committee at Pontificia Universidad Católica de Chile (Protocol 201223001). The patients/participants provided their written informed consent to participate in this study.

## Author contributions

CP-L, JG, MD, and CM-L: conceptualization. SC, MD, CM-L, CP-L, and JG: experimental design. SC and CP-L: data collection. CP-L, LL, and YJ: analysis. CP-L and YJ: original draft preparation. CP-L, LL, JG, and CM-L: editing. All authors reviewed the manuscript.
