# Supplementary material for: Eating contexts determine the efficacy of nutrient warning labels to promote healthy food choices
Source: Front Nutr. 2023 Jan 6;9:1026623. doi: 10.3389/fnut.2022.1026623 (PMC9852898; doi:10.3389/fnut.2022.1026623)
Supplement: Supplementary file 2 [file Data_Sheet_2.docx]

**Supplementary Material**

**Supplementary Tables and Figures**

**Table S1. Detailed Experimental workflow**. This table details the steps for the experimental procedure.

| Experimental phase | Steps | Details |
| --- | --- | --- |
| Screening | 1. Questionnaire | Participants completed a screening questionnaire including body weight, height, age, variation of body weight in the last two months, dietary lifestyle, alcohol, and tobacco use. |
| First session | 1. Questionnaire | Participants completed a questionnaire containing the same questions as the screening phase. |
|  | 1. Time of last meal. | Participants recorded their time of last meal |
|  | 1. Sleep Stanford scale (SSS) | Participants completed the SSS |
|  | 1. Hunger and appetite questions | Participants completed for questions about hunger and appetite using a VAS scale from 0 to 100 |
|  | 1. Adult eating behavior questionnaire (AEBQ) | Participants completed the AEBQ |
|  | 1. Food image recognition | Participants answered a yes/no question indicating whether they recognized 94 food images. |
|  | 1. Health, liking and wanting ratings | Participants rated all 94 images for healthiness, liking, and wanting using a VAS scale from 0 to 100 |
| Second session | 1. Time of last meal. | Participants recorded their time of last meal |
|  | 1. Sleep Stanford scale (SSS) | Participants completed the SSS |
|  | 1. Hunger and appetite questions | Participants completed for questions about hunger and appetite using a VAS scale from 0 to 100 |
|  | 1. Training task | Participants were asked to choose the desk item between images of a desk and a kitchen item |
|  | 1. Choose food images without displaying NWL | Participants had to choose one food image from 90 pairs within a randomized context sequence (typical, healthy, unrestricted) |
|  | 1. Choose images displaying NWL | Same procedure as before but displaying NWL for each image. |

**Table S2. Screening questionnaire.** This table shows the questions included in the screening questionnaire in the original Spanish and its English translation.

| **Question Number** | **Spanish** | **English** |
| --- | --- | --- |
| 1 | ¿Tiene usted alguna enfermedad física o psicológica? (responda sí o no):  Si ha respondido SI, ¿Cuál (es)?: | Do you have a physical or mental illness? (Answer yes or no). If you answered yes, please describe. |
| 2 | ¿Presenta en la actualidad alguna discapacidad o problema visual que le impida distinguir imágenes proyectadas en una pantalla? (responda sí o no):  Si ha respondido SI, ¿Cuál (es)?: | Do you have any disability or visual impairment that prevents you from distinguishing images shown on a screen? (Answer yes or no). If you answered yes, please describe. |
| 3 | ¿Ha sufrido variaciones de su peso en los últimos tres meses? (responda sí o no):  Si ha respondido SI, ¿Cuántos kilogramos ha perdido o ganado?: | Has your body weight changed more than 3 kg in the last three months? (Answer yes or no). If you answered yes, please describe. |
| 4 | ¿Usted bebe alcohol? (responda sí o no):  Si ha respondido SI, ¿Cuántos vasos a la semana? | Do you drink alcohol? (Answer yes or no). If you answered yes, please indicate how many servings per week. |
| 5 | ¿Usted fuma? (responda sí o no):  Si ha respondido SI, ¿Cuántos al día?: | Do you smoke? (Answer yes or no). If you answered yes, please indicate how many times per day. |
| 6 | ¿Usa algún medicamento, suplemento, vitaminas o minerales? (responda sí o no):  Si ha respondido SI, ¿Cuál (es)?: | Do you use any prescription, supplements, vitamins, or minerals? (Answer yes or no). If you answered yes, please describe. |
| 7 | ¿Está usted con algún régimen alimentario o tratamiento para subir o bajar de peso? (responda sí o no): | Are you currently with any dietary intervention or treatment to gain or lose weight? (Answer yes or no). |
| 8 | ¿Tiene alguna intolerancia, alergia alimentaria? (responda sí o no):  Si ha respondido SI, ¿cuál? | Do you have any intolerance or food allergies? (Answer yes or no). If you answered yes, please describe. |
| 9 | ¿Es vegetariano, tiene alguna restricción alimentaria o algún alimento que no sea de su agrado? (responda sí o no):  Si ha respondido SI, ¿cuál? | Do you follow a vegetarian diet, have any food restrictions, or any food that is not to your liking?  (Answer yes or no). If you answered yes, please describe. |
| 10 | ¿Participa de algún tipo de ejercicio físico o deporte? No incluya caminatas (responda sí o no):  Si ha respondido SI, ¿cuál(es) y cuántas veces por semana? | Do you participate in any type of physical activity? Do not include leisure walks (Answer yes or no). If you answered yes, please describe and indicate times per week. |
| 11 | ¿Está embarazada o en periodo de lactancia? | Are you pregnant or breastfeeding? (Answer yes or no). |

**Table S3. Visual Analog Scales (VAS) Questions**. All VAS questions used in the study in the original Spanish and their English version.

| **Question Number** | **Language** | **Question Class** | **Question** | **Left Anchor** | **Right Anchor** |
| --- | --- | --- | --- | --- | --- |
| 1 | Spanish | Apetito | ¿Qué tan hambriento está usted ahora? | Nada | Extremadamente |
|  | English | Appetite | ¿How hungry are you right now? | Not at all | Extremely |
| 2 | Spanish | Apetito | ¿Qué tan satisfecho está usted ahora? | Nada | Extremadamente |
|  | English | Appetite | ¿How satisfied are you right now? | Not at all | Extremely |
| 3 | Spanish | Apetito | ¿Cuánto es su deseo de consumir alimentos ahora? | Nada | Mucho |
|  | English | Appetite | ¿How much do you want to eat food right now? | Not at all | A lot |
| 4 | Spanish | Apetito | ¿Cuánto piensa usted que podría comer en este momento? | Nada | Mucho |
|  | English | Appetite | ¿How much food would you like to eat right now? | Not at all | A lot |
| 5 | Spanish | Deseo | ¿Cuánto desea Ud. consumir este alimento en este momento? | Nada en absoluto | Extremadamente |
|  | English | Want | ¿How much do you want to eat this food right now? | Not at all | Extremely |
| 6 | English | Like | ¿How much do you like this food? | Not at all | Extremely |
|  | Spanish | Gusto | ¿Cuánto le gusta a Ud. este alimento? | Nada en absoluto | Extremadamente |
| 7 | Spanish | Salud | ¿Qué tan saludable considera Ud. este alimento? | Nada en absoluto | Extremadamente |
|  | English | Health | ¿How healthy would you consider this food? | Not at all | Extremely |

**Table S4. ANOVA results for effects of context (Healthy, Typical, Unrestricted) on nutritional and calorie content of food images.** Detailed ANOVA output for models of effect of number of NWL and context on calories, fat, sugar, and salt content of food images used in different contexts.

| **Dependent Variable** | **Model Term** | **df** | **SS** | **MS** | **F** | **P** |
| --- | --- | --- | --- | --- | --- | --- |
| Sugar per 100 g | Context | 2 | 683.76 | 341.88 | 1.01 | 0.37 |
|  | NWLnumber | 3 | 10650.16 | 3550.05 | 10.47 | 0.00 |
|  | Context:NWL_number | 6 | 2051.68 | 341.95 | 1.01 | 0.43 |
|  | Residuals | 82 | 27804.10 | 339.07 |  |  |
| Saturated Fat per 100 g | Context | 2 | 66.55 | 33.27 | 1.66 | 0.20 |
|  | NWL_number | 3 | 1115.82 | 371.94 | 18.52 | 0.00 |
|  | Context:NWL_number | 6 | 90.99 | 15.16 | 0.76 | 0.61 |
|  | Residuals | 82 | 1646.72 | 20.08 |  |  |
| Kcal per 100 g | Context | 2 | 1425.63 | 712.82 | 0.08 | 0.93 |
|  | NWL_number | 3 | 1980053.39 | 660017.80 | 70.94 | 0.00 |
|  | Context:NWL_number | 6 | 10706.61 | 1784.44 | 0.19 | 0.98 |
|  | Residuals | 82 | 762902.36 | 9303.69 |  |  |
| Sodium per 100 mg | Context | 2 | 116543.12 | 58271.56 | 0.71 | 0.49 |
|  | NWL_number | 3 | 1731973.59 | 577324.53 | 7.08 | 0.00 |
|  | Context:NWL_number | 6 | 206007.50 | 34334.58 | 0.42 | 0.86 |
|  | Residuals | 82 | 6687231.92 | 81551.61 |  |  |

**Table S5. Detailed output of mixed-model logistic regression for choice made in the absence of NWL.** Output of mixed-model logistic regression for probability of a healthy food choice made without showing NWL. ΔLike: Difference in like rating between images, ΔHealth: Difference in health rating between images.

| **Model Term** | **Estimate ± SE** | **Statistic** | **p-value** |
| --- | --- | --- | --- |
| (Intercept) | 0.509 ± 0.053 | 9.69 | <0.01 |
| Trial Number | -0.001 ± 0.001 | -0.84 | 0.399 |
| Age | -0.018 ± 0.041 | -0.45 | 0.652 |
| BMI | 0.084 ± 0.039 | 2.12 | 0.034 |
| SSS | -0.009 ± 0.037 | -0.24 | 0.808 |
| Physical Exercise | -0.031 ± 0.041 | -0.78 | 0.438 |
| AEBQ score | 0.023 ± 0.041 | 0.57 | 0.568 |
| Appetite score | -0.077 ± 0.043 | -1.78 | 0.075 |
| Time of test | 0.036 ± 0.039 | 0.94 | 0.348 |
| Time of Last Meal | 0.021 ± 0.044 | 0.48 | 0.633 |
| Sex | 0.046 ± 0.039 | 1.20 | 0.231 |
| ΔHealth | 0.039 ± 0.001 | 68.19 | <0.01 |
| Healthy vs Typical Context | 0.482 ± 0.03 | 15.80 | <0.01 |
| Unrestricted vs Typical Context | -0.087 ± 0.028 | -3.12 | <0.01 |
| ΔLike | -0.006 ± 0.001 | -10.39 | <0.01 |
| ΔHealth:Healthy Context vs Typical | -0.001 ± 0.001 | -1.30 | 0.194 |
| ΔHealth:Unrestricted Context vs Typical | 0 ± 0.001 | -0.40 | 0.689 |
| ΔLike:Healthy Context vs Typical | -0.003 ± 0.001 | -3.24 | <0.01 |
| ΔLike:Unrestricted Context vs Typical | 0.002 ± 0.001 | 2.22 | 0.026 |

**Table S6. Detailed output of effects of eating context and choice made in the absence of NWL on AUC and RT.** ANOVA Table (Wald Type III Test Estimates) of linear mixed model for adjusted effects of eating context on AUC and RT during food choice made without showing NWL

| **Term** | **AUC** | **RT** |
| --- | --- | --- |
| (Intercept) | 381.1 (df=1), P<0.01 | 3704.7 (df=1), P<0.01 |
| Trial Number | 1.03 (df=1), P=0.31 | 1167.5 (df=1), P<0.01 |
| Age | 1.6 (df=1), P=0.206 | 1.03 (df=1), P=0.311 |
| BMI | 0.58 (df=1), P=0.447 | 1.68 (df=1), P=0.195 |
| SSS | 1.62 (df=1), P=0.203 | 0.29 (df=1), P=0.593 |
| Physical Exercise | 0 (df=1), P=0.993 | 1.88 (df=1), P=0.17 |
| AEBQ score | 3.53 (df=1), P=0.06 | 0.01 (df=1), P=0.916 |
| Appetite Score | 1.27 (df=1), P=0.26 | 0.04 (df=1), P=0.844 |
| Time of test | 0.62 (df=1), P=0.432 | 0.34 (df=1), P=0.561 |
| Time from last meal | 0.1 (df=1), P=0.753 | 0.2 (df=1), P=0.656 |
| Sex | 0.02 (df=1), P=0.892 | 7 (df=1), P<0.01 |
| Choice (Healthy/Unhealthy) without NWL | 510.61 (df=1), P<0.01 | 379.44 (df=1), P<0.01 |
| Eating Context | 1.91 (df=2), P=0.384 | 22.04 (df=2), P<0.01 |
| Choice (Healthy/Unhealthy) without NWL: Eating Context | 10.52 (df=2), P<0.01 | 242.47 (df=2), P<0.01 |

**Table S7. Detailed output of model for change in AUC and RT during choice made when NWL were shown relative to choices made in the absence of NWL.** ANOVA Table (Wald Type III Test Estimates) of linear mixed model for adjusted effects of eating context on change in AUC and RT during food choice when NWL were shown

| **Term** | **ΔAUC** | **ΔRT** |
| --- | --- | --- |
| **term** | **AUC** | **RT** |
| (Intercept) | 303.6 (df=1), P<0.01 | 2178.15 (df=1), P<0.01 |
| AUC or RT during choice without NWL | 13538.05 (df=1), P<0.01 | 16940.73 (df=1), P<0.01 |
| Trial Number | 4.76 (df=1), P=0.029 | 256.05 (df=1), P<0.01 |
| Age | 0.41 (df=1), P=0.522 | 1.83 (df=1), P=0.176 |
| BMI | 2.37 (df=1), P=0.124 | 1.32 (df=1), P=0.251 |
| SSS | 0.72 (df=1), P=0.395 | 0 (df=1), P=0.963 |
| Physical Exercise | 0.55 (df=1), P=0.46 | 3.77 (df=1), P=0.052 |
| AEBQ score | 4.87 (df=1), P=0.027 | 0.04 (df=1), P=0.838 |
| Appetite Score | 1.63 (df=1), P=0.201 | 0.54 (df=1), P=0.464 |
| Time of test | 1.07 (df=1), P=0.301 | 0.79 (df=1), P=0.375 |
| Time from last meal | 0.07 (df=1), P=0.798 | 0.03 (df=1), P=0.858 |
| Sex | 0.23 (df=1), P=0.631 | 6.63 (df=1), P=0.01 |
| Decision Path | 155.63 (df=3), P<0.01 | 506.44 (df=3), P<0.01 |
| Context | 9.2 (df=2), P=0.01 | 25.41 (df=2), P<0.01 |
| Decision Path:Context | 14.37 (df=6), P=0.026 | 140.59 (df=6), P<0.01 |

**Figure S1**. Workflow for participant selection

**Figure S2**. Nutrient and calorie density of food images in each context. Y-axis shows mean and standard error of the mean

**Figure S3**. Adjusted estimates for health, like and want ratings based on demographic characteristics. Plots A, E, and I show health, like, and want ratings, respectively, for normal weight participants that declared to not engage in physical activity. Plots B, F, and J show health, like, and want ratings, respectively, for normal weight participants that declared to engage in physical activity. Plots C, G, and K show health, like, and want ratings, respectively, for overweight participants that declared to not engage in physical activity. Plots D, H, and L show health, like, and want ratings, respectively, for overweight participants that declared to engage in physical activity. All plots show data separated by biological sex. For all plots, y-axis shows mean and standard error of the mean

**Figure S4**. Effect of declared label use on the probability of a healthy food choice in the presence of NWL. Decision paths separate plots. (A) When an unhealthy choice was made without NWL. (B) When a healthy choice was made without NWL. Brackets indicate p<0.05 for pairwise comparisons between eating contexts within the declared type of label use.

**Figure S5.** Effect of increase in the absolute difference in nutritional warning labels (ΔNWL) on the probability of a healthy food choice when participants declared to (A) count the number of NWL, (B) not use NWL during choice, (C) read the information displayed in the NWL. Brackets indicate p<0.05 for pairwise comparisons between DNWL within eating context for each declared type of label use
